# The duration of intra-abdominal hypertension strongly predicts outcomes for the critically ill surgical patients: a prospective observational study

**DOI:** 10.1186/s13017-015-0016-7

**Published:** 2015-05-30

**Authors:** Kyu-Hyouck Kyoung, Suk-Kyung Hong

**Affiliations:** Department of Surgery, Ulsan University Hospital, University of Ulsan College of Medicine, 877 Bangeojinsunhwando-ro, Dong-gu, Ulsan, Republic of Korea; Division of Trauma and Surgical Critical Care, Department of Surgery, Asan Medical Center, University of Ulsan College of Medicine, 388-1 Pungnap-dong, Songpa-gu, Seoul, Republic of Korea

**Keywords:** Severe sepsis, Intra-abdominal hypertension, Intra-abdominal pressure, Enteral feeding, abdominal perfusion pressure

## Abstract

**Introduction:**

Intra-abdominal hypertension (IAH) is associated with morbidity and mortality in critically ill patients. The present study analyzed the clinical significance of IAH in surgical patients with severe sepsis.

**Methods:**

This was a prospective study carried out in the surgical intensive care unit (SICU). Intra-abdominal pressure (IAP) was measured three times a day via a urinary catheter filled with 25 mL of saline. IAH was defined as an IAP ≥ 12 mmHg, and the peak IAP was recorded as the IAP for the day. Data were analyzed in terms of IAH development and the IAH duration.

**Results:**

Of the 46 patients enrolled in the study, 42 developed IAH while in the SICU. The development of IAH aggravated the clinical outcomes; such as longer SICU stay, requirement of ventilator support, and delayed initiation of enteral feeding (EF). The IAH duration showed a significant correlation with pulmonary, renal, and cardiovascular function, and enteral feeding. The IAH duration was an independent predictor of 60-day mortality (odds ratio: 1.196; *p* = 0.014).

**Conclusions:**

The duration of IAH is a more important prognostic factor than the development of IAH; thus every effort should be made to reduce the IAH duration in critically ill patients.

**Trial registration:**

NCT01784458

## Introduction

Intra-abdominal pressure (IAP) is defined as the steady-state pressure within the abdominal cavity bounded by the abdominal muscles and diaphragm [1]. It is affected by body weight, posture, tension of abdominal muscles, and movement of the diaphragm [2–4]. The World Society of the Abdominal Compartment Syndrome (WSACS) has published a grading system for intra-abdominal hypertension (IAH), with IAH defined as an IAP ≥12 mmHg, and abdominal compartment syndrome as an IAP ≥ 20 mmHg combined with the failure of more than one organ [1, 5]. Ever since then, WSACS have updated consensus definitions and clinical practice guidelines for the patients with IAH [6]. The prevalence of IAH on admission to the intensive care unit (ICU) ranges from 31 to 58.8 % [4, 7, 8], and the incidence increases with the length of ICU stay. Clinical conditions that increase IAH include blood and ascites in the peritoneal cavity, bowel distension and edema [4, 9], high-volume resuscitation and massive transfusion, damage control surgery in traumatic patients, excessive tension after abdominal closure, postoperative ileus, echar in burn patients [10, 11], and hemodilution [12]. IAH causes not only abdominal organ dysfunction by decreasing the abdominal perfusion pressure (APP) [13–15] but also cardiopulmonary dysfunction [16], which increases both morbidity and mortality [17].

IAH has been increasingly recognized in the critically ill patients. However, the duration of IAH has not been under consideration. The aim of the present study was to investigate the influence of IAH development and its duration on the clinical course and outcome of critically ill surgical patients with severe sepsis.

## Materials and methods

### Study design and patients

The study was a prospective observational study in surgical ICU of an academic tertiary care hospital. Patients at least 18 years of age, who admitted for severe sepsis were enrolled within 24 h of admission to the ICU. Patients with urinary tract injury or therapeutic open abdomen were excluded. All subjects provided informed consent. A total of 48 patients admitted to the ICU from March 2009 to October 2009 met the inclusion criteria, of which two were excluded who refused to participate in the study. Overall, 46 patients were enrolled. The study protocols were approved by the Institutional Review Board of Asan Medical Center and registered at http://ClinicalTrials.gov under the number NCT01784458.

### Definitions

Severe sepsis was defined as a sepsis with a failure of more than one organ due to sepsis, an arterial blood lactate concentration of at least 4 mmol/L, or hypotension (with a systolic blood pressure < 90 mmHg).

### Measurements and treatment

IAP was measured using a urinary catheter at the level of the mid-axillary line on the iliac crest with the patient supine. IAP was expressed as mmHg. A 3-lumen urinary catheter was inserted into the bladder. After the urinary drainage lumen was clamped, 25 ml of saline was injected through the irrigation lumen to prevent contamination. IAP was measured three times per day while the patient was in the ICU, with the highest reading recorded as the value for that day. The Acute Physiology and Chronic Health Evaluation (APACHE II) score was recorded every 24 h. Resuscitation was performed according to goal-directed guidelines [18, 19].

### Statistical analysis

Statistical analyses were performed using SPSS 21 for windows (SPSS Inc. Chicago, IL). The Chi-square test or Fisher’s exact test were used to compare categorical variables and the Mann–Whitney *U* test was used to compare continuous variables. Correlation analysis was performed using Spearman’s rank correlation coefficient. Statistical significance was set at *p* < 0.05.

## Results

### Baseline characteristics

On admission day, 33 patients (71.7 %) had IAH. Mean IAP was 17.3 ± 5.5 mmHg. During ICU stay, IAH developed in 42 (91.3 %) patients at 1.4 ± 1.0 days after ICU admission. The incidence of IAH was higher in patients with peritonitis [25 (59.5 %) vs. 0 (0 %), *p* = 0.037] and in those who underwent laparotomy [37 (88.1 %) vs. 1 (25 %), *p* = 0.013]. The APACHE II score (22.0 ± 6.1 vs. 14.0 ± 6.2, *p* = 0.030) and total fluid administered (5,065 ± 1,814 ml vs. 2,657 ± 927 ml, *p* = 0.007) was significantly high in the IAH group. There were no significant differences in other parameters between IAH group and non-IAH group on admission day to the ICU (Table 1).

**Table 1:** Baseline characteristics of the study patients upon admission to the intensive care unit

Variable	IAH (n = 42)	Non-IAH (n = 4)	*P*-value
Age (years)	64.9 ± 12.0	63.8 ± 8.7	0.585
Gender			>0.99
male	32 (76.2)	3 (75.0)	
Female	10 (23.8)	1 (25.0)	
Causes of sepsis			0.037
Peritonitis	25 (59.5)	0	
non- peritonitis	17 (40.5)	4 (100)	
abdominal organ infection	11 (26.2)	3 (75)	
Pneumonia	3 (7.1)	1(25)	
Others	3 (7.1)	0	
Laparotomy			0.013
laparotomy	37 (88.1)	1 (25.0)	
non-laparotomy	5 (11.9)	3 (75.0)	
APACHE II Score	22.0 ± 6.1	14.0 ± 6.2	0.030
Total fluid (mL/day)	5065 ± 1814	2657 ± 927	0.007
Urine output (mL/day)	1615 ± 1148	1243 ± 1011	0.560
RBC transfusion (units)	0.7 ± 1.8	1.3 ± 1.5	0.395
White blood cell (× 10^3^/mm^3^)	14.4 ± 12.0	9.6 ± 4.7	0.721
Hematocrit (%)	28.4 ± 5.2	27.1 ± 7.3	0.374
Platelet (× 10^3^/mm^3^)	120.2 ± 119.0	63.0 ± 26.8	0.117
Prothrombin time (%)	45.5 ± 18.9	46.7 ± 15.6	0.638
Total bilirubin (mg/dL)	1.9 ± 1.4	3.8 ± 3.0	0.127
Blood urea nitrogen^1^ (mg/dL)	28.2 ± 16.9	34.3 ± 21.5	0.515
Creatinine^1^ (mg/dL)	1.6 ± 1.0	1.7 ± 1.0	0.829
Lactate (mmol/L)	3.9 ± 2.4	3.7 ± 1.1	0.836
PaO_2_/FiO_2_ Ratio	151.9 ± 69.8	212.5 ± 65.7	0.099

^1^Two patients with chronic renal failure on hemodialysis were excluded from the IAH group

### Clinical outcomes according to intra-abdominal hypertension

The length of ICU stay (17.3 ± 13.2 days vs. 2.5 ± 2.4 days, *p* = 0.002) and length of hospital stay (42.6 ± 27.6 days vs. 16.0 ± 2.4 days, *p* = 0.003) were longer in the IAH group. Patients with the IAH had an increased requirement for mechanical ventilation [38 (90.5 %) vs. 1 (25 %), *p* = 0.009] and duration of ventilatory support (13.1 ± 13.0 day vs. 1.0 ± 2.0 day, *p* = 0.007). Initiation of enteral feeding (EF) was delayed (8.9 ± 7.5 day vs. 2.3 ± 1.9 day, *p* = 0.019) in the IAH group. Renal replacement therapy (RRT) and mortality did not show significant differences (Table 2).

**Table 2:** Clinical outcomes according to intra-abdominal hypertension

Variable	IAH (n = 42)	Non-IAH (n = 4)	*P*-value
Mortality			
30-Day mortality	5 (11.9)	0	>0.99
60-Day mortality	13 (31.0)	0	0.313
Length of ICU Stay (days)	17.3 ± 13.2	2.5 ± 2.4	0.002
Length of hospital Stay (days)	42.6 ± 27.6	16.0 ± 2.4	0.003
Mechanical ventilation	38 (90.5)	1 (25.0)	0.009
Duration of ventilatory support (days)	13.1 ± 13.0	1.0 ± 2.0	0.007
Renal replacement therapy^1^	15 (37.5)	0	0.282
Duration of RRT^1^ (days)	6.5 ± 10.9	0	0.239
Vasopressor treatment	36 (85.7)	3 (75.0)	0.496
Inotropic treatment	13 (31.0)	0	0.313
Initiation of enteral feeding^2^ (days)	8.9 ± 7.5	2.3 ± 1.9	0.019

^1^Two patients with chronic renal failure on hemodialysis were excluded from the IAH group

^2^Two patients in the IAH group (who did not try enteral feeding) were excluded

### Clinical effect of the duration of intra-abdominal hypertension

**Fig. 1:**
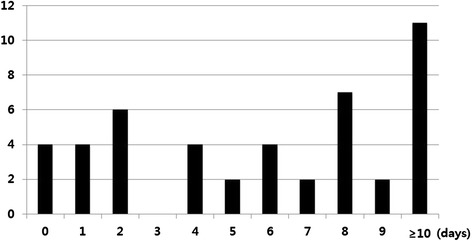
Distribution of patients by duration of intra-abdominal hypertension

**Fig. 2:**
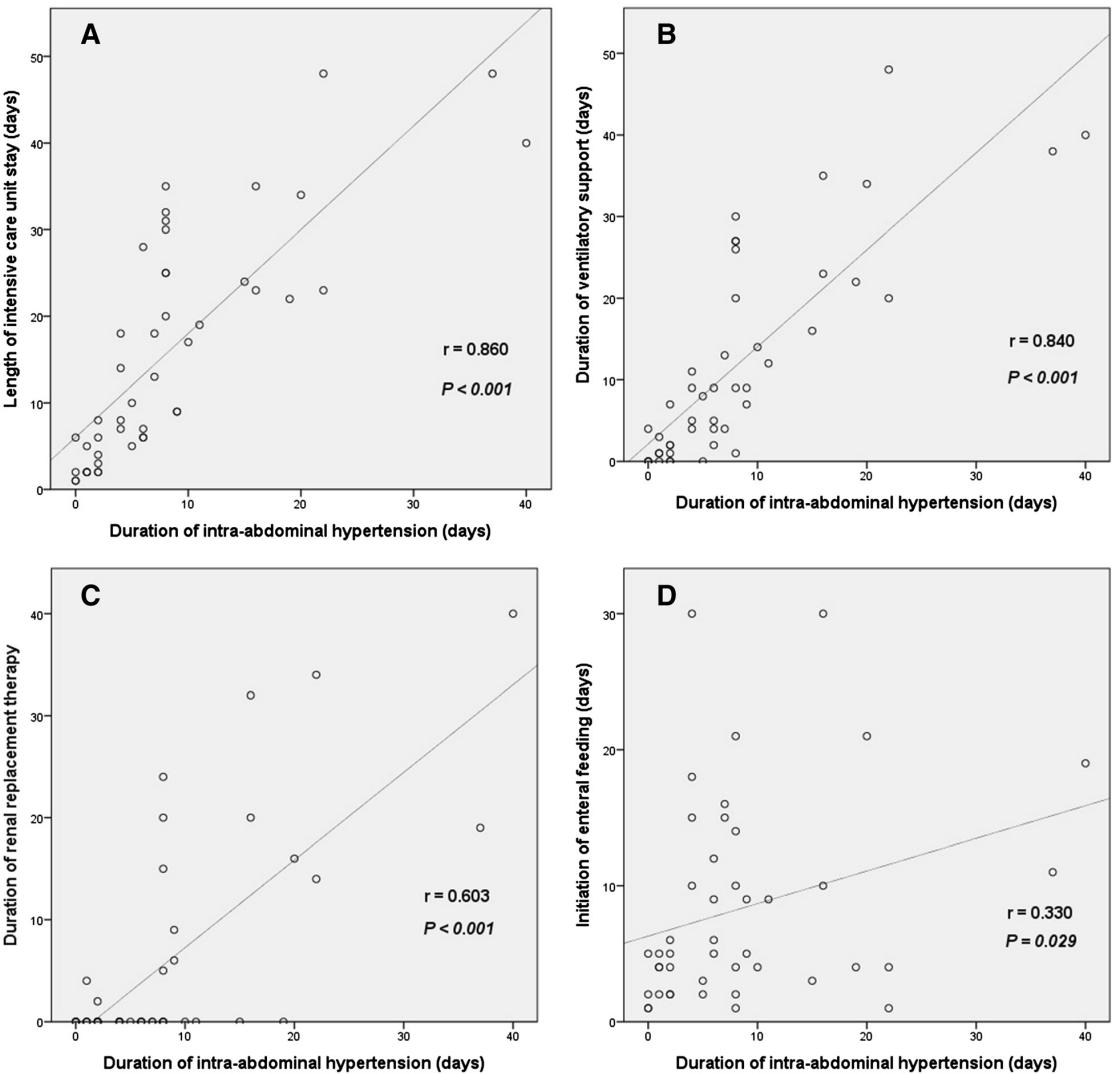
Clinical course according to duration of intra-abdominal hypertension. Correlation between duration of intra-abdominal hypertension and **a** length of intensive care unit stay, **b** duration of ventilatory support, **c** duration of renal replacement therapy, **d** initiation of enteral feeding. Two patients with chronic renal failure on hemodialysis were excluded on Fig. 2c. Two patients who did not try enteral feeding were excluded on Fig. 2d

The duration of IAH had more impact on outcomes than the development of IAH. Distribution of IAH duration is demonstrated on Fig. 1. There were significant increases in terms of the length of ICU stay (r = 0.860, *p* < 0.001), duration of mechanical ventilation (r = 0.840, *p* < 0.001), duration of RRT (r = 0.603, *p* < 0.001), and initiation of EF (r = 0.330, *p* = 0.029) according to increase of IAH duration (Fig. 2).

### Comparison of survivors and non-survivors

There were no significant differences in any of the study variables in terms of 30-day mortality. However, univariate analysis showed that the duration of IAH (*p* = 0.001), the initial APACHE II score (*p* = 0.021), and peritonitis (*p* = 0.010) were significantly associated with 60-day mortality (Table 3). Multivariate logistic regression analysis identified the duration of IAH as an independent predictor of 60 day-mortality (odds ratio: 1.196; 95 % confidence interval: 1.037–1.380; *p* = 0.014) (Table 4).

**Table 3:** Univariate analysis on the predictors of 60-Day mortality

Variable	Survivors (n = 33)	Non-survivors (n = 13)	*P*-value
Age (years)	64.1 ± 12.7	66.5 ± 8.6	0.517
Initial APACHE II Score	19.9 ± 5.9	24.7 ± 6.8	0.021
Peritonitis	14 (42.4)	11 (84.6)	0.010
Development of IAH	29 (87.9)	13 (100)	0.313
Duration of IAH (days)	5.2 ± 4.1	16.3 ± 12.2	0.001

**Table 4:** Multivariate analysis on predictors of 60-day mortality

Variable	Odds ratio	95 % Confidence interval	*P*-value
Initial APACHE II Score	1.073	0.917–1.254	0.381
Peritonitis	6.072	0.814–45.278	0.078
Duration of IAH	1.196	1.037–1.380	0.014

## Discussion

IAP has been measured since the 19th century. Ever since then, its importance has been recognized recently. Since the mid-1990s it was known that IAH could develop without abdominal trauma [20] and numerous studies have measured IAP, examined its clinical outcomes, and classifications.

The prevalence of IAH depends on the patient population. IAH was present in 54.4 % of medical ICU and 65 % of surgical ICU patients [7]. We found a quite high prevalence of IAH (91.3 %) in critically ill surgical patients. The previous study demonstrated that sepsis was the predominant cause of IAH [21], and the population of this study composed of the patients with severe sepsis might make such deviation.

This study was to introduce the clinical effects of time-dependence of IAH. The effect off IAH in organ dysfunction involves a myriad of pathologic change. Essentially, the concept of IAH has been established in the area of trauma and experts have been recommended damage control laparotomy and open abdomen to correct physiologic stress in trauma and acute general surgical patients [22]. The main mechanism of organ dysfunction by IAH is suggested as reduction of APP and direct pressure effect on other compartment [17]. Most of previous studies have focused on the effect of IAH. However, the duration of IAH is more important as an outcome prognostic factor than a just presence of IAH. It could be explained that prolonged IAH would accumulate risk for organ failure and worsen the outcomes. This study identified the phenomenon that persistent IAH aggravated organ failure and increased affected mortality independently.

Patients with IAH tend to need more mechanical ventilation and have more difficulty in weaning, both of which represent respiratory organ failure. The transmission of IAH to thorax has a worse impact on the respiratory system. The major reason is a reduction of the functional residual capacity caused by cephalad displacement of the diaphragm in response to unopposed intra-abdominal pressure. In addition, reduction of chest wall compliance cause by IAH is leading to atelectasis [23]. Therefore, patients with IAH need a different ventilator strategy and more specific treatment considering IAH such as positive end-expiratory pressure against IAP [24]. Moreover, persistent IAH can worsen the respiratory mechanics more seriously.

The adverse effects of IAH on abdominal organs have been demonstrated on the basis of renal function in postoperative surgical patients [21, 25]. Acute kidney injury is common consequences of IAH. The detrimental effect of IAH on the kidney is closely related to renal blood flow. IAH lead to significant intrarenal venous congestion and to lower the filtration gradient, which represents the difference between glomerular filtration and proximal tubular pressures [26, 27]. With persistent IAH, not only direct effect of intra-abdominal pressure but also reduction of cardiac output and elevated level of catecholamine, rennin, angiotensin, and inflammatory cytokines may also come into play, further worsening renal function.

Elevated IAP also reduces blood flow to abdominal viscera [28, 29]. Splanchnic ischemia impairs subsequent intestinal barrier function and gastrointestinal motility. Early enteral nutrition is the preferred strategy for feeding the critically ill patients. However, it is not always possible to initiate EN in critically ill patients. Elevated IAP can be a big obstacle to feed early the critically ill patients. In addition, it is very hard to determine the tolerance of enteral feeding in patients with IAH. Therefore, the results presented herein suggest that IAH and its duration may provide important clues for clinicians to make decisions about whether to proceed with EF. As a result, persistent IAH delays enteral feeding in various reasons.

In addition to IAH development, sustained IAH reduced the chances of recovery and forces patients into a vicious cycle. The results of the present study show that the IAH duration is a more important clinical factor than the development of IAH.

## Conclusions

There is a strong relationship ‘risk accumulation’ between duration of IAH and organ dysfunction. Persistent elevations of IAH are aggravating clinical outcomes including organ failure and mortality. Therefore early recognition and prompt intervention, including surgical intervention if necessary are essential to improve patients’ outcomes.

## Abbreviations

IAP: Intra-abdominal pressure
WSACS: World Society of the Abdominal Compartment Syndrome
IAH: Intra-abdominal hypertension
ICU: Intensive care unit
APP: Abdominal perfusion pressure
APACHE II: Acute Physiology and Chronic Health Evaluation
EF: Enteral feeding
RRT: Renal replacement therapy
RBC: Red blood cell

## References

[1] Malbrain ML, Cheatham ML, Kirkpatrick A, Sugrue M (2006). Results from the International Conference of Experts on Intra-abdominal Hypertension and Abdominal Compartment Syndrome. I. Definitions. Intensive Care Med.

[2] Pelosi P, Croci M, Ravagnan I, Cerisara M, Vicardi P, Lissoni A (1997). Respiratory system mechanics in sedated, paralyzed, morbidly obese patients. J Appl Physiol.

[3] Hering R, Wrigge H, Vorwerk R, Brensing KA, Schröder S, Zinserling J (2001). The effects of prone positioning on intra-abdominal pressure and cardiovascular and renal function in patients with acute lung injury. Anesth Analg.

[4] Malbrain ML, Chiumello D, Pelosi P, Bihari D, Innes R, Ranieri VM (2005). Incidence and prognosis of intra-abdominal hypertension in a mixed population of critically ill patients: a multiple-center epidemiological study. Crit Care Med.

[5] Cheatham ML, Malbrain ML, Kirkpatrick A, Sugrue M (2007). Results from the International Conference of Experts on Intra-abdominal Hypertension and Abdominal Compartment Syndrome. II. Recommendations. Intensive Care Med.

[6] Kirkpatrick AW, Roberts DJ, De Waele J, Jaeschke R, Malbrain ML, De Keulenaer B (2013). Intra-abdominal hypertension and the abdominal compartment syndrome: updated consensus definitions and clinical practice guidelines from the World Society of the Abdominal Compartment Syndrome. Intensive Care Med.

[7] Vidal MG, Ruiz Weisser J, Gonzalez F, Toro MA, Loudet C (2008). Incidence and clinical effects of intra-abdominal hypertension in critically ill patients. Crit Care Med.

[8] Malbrain ML, Chiumello D, Pelosi P, Wilmer A (2004). Prevalence of intra-abdominal hypertension in critically ill patients: a multicentre epidemiological study. Intensive Care Med.

[9] Goldman RK, Mullins RJ (2003). Mechanism of acute ascites formation after trauma resuscitation. Arch Surg.

[10] Mahajna A, Mitkal S, Krausz MM (2008). Postoperative gastric dilatation causing abdominal compartment syndrome. World J Emerg Surg.

[11] De Waele JJ, Hoste E, Blot SI, Decruyenaere J, Colardyn F (2005). Intra-abdominal hypertension in patients with severe acute pancreatitis. Crit Care.

[12] Czajkowski M, Dabrowski W (2006). Changes in intra-abdominal pressure during CABG with normovolemic hemodilution. Med Sci Monit.

[13] Diebel LN, Wilson RF, Dulchavsky SA, Saxe J (1992). Effect of increased intra-abdominal pressure on hepatic arterial, portal venous, and hepatic microcirculatory blood flow. J Trauma.

[14] Diebel LN, Dulchavsky SA, Brown WJ (1997). Splanchnic ischemia and bacterial translocation in the abdominal compartment syndrome. J Trauma.

[15] De Laet IE, Ravyts M, Vidts W, Valk J, De Waele JJ (2008). Current insights in intra-abdominal hypertension and abdominal compartment syndrome: open the abdomen and keep it open!. Arch Surg.

[16] Ridings PC, Bloomfield GL, Blocher CR, Sugerman HJ (1995). Cardiopulmonary effects of raised intra-abdominal pressure before and after intravascular volume expansion. J Trauma.

[17] Cheatham ML, White MW, Sagraves SG, Johnson JL, Block EF (2000). Abdominal perfusion pressure: a superior parameter in the assessment of intra-abdominal hypertension. J Trauma.

[18] Rivers E, Nguyen B, Havstad S, Ressler J, Muzzin A (2001). Early goal-directed therapy in the treatment of severe sepsis and septic shock. N Engl J Med.

[19] Dellinger RP, Carlet JM, Masur H, Gerlach H, Calandra T (2004). Surviving Sepsis Campaign guidelines for management of severe sepsis and septic shock. Crit Care Med.

[20] Greenhalgh DG, Warden GD (1994). The importance of intra-abdominal pressure measurements in burned children. J Trauma.

[21] Sugrue M, Jones F, Deane SA, Bishop G, Bauman A, Hillman K (1999). Intra-abdominal hypertension is an independent cause of postoperative renal impairment. Arch Surg.

[22] Godat L, Kobayashi L, Costantini T, Coimbra R (2013). Abdominal damage control surgery and reconstruction: world society of emergency surgery position paper. World J Emerg Surg.

[23] Pelosi P, Quintel M, Malbrain ML (2007). Effect of intra-abdominal pressure on respiratory mechanics. Acta Clin Belg Suppl.

[24] Regli A, Chakera J, De Keulenaer BL, Roberts B, Noffsinger B, Singh B (2012). Matching positive end-expiratory pressure to intra-abdominal pressure prevents end-expiratory lung volume decline in a pig model of intra-abdominal hypertension. Crit Care Med.

[25] Sugrue M, Buist MD, Hourihan F, Deane S, Bauman A, Hillman K (1995). Prospective study of intra-abdominal hypertension and renal function after laparotomy. Br J Surg.

[26] Dalfino L, Tullo L, Donadio I, Malcangi V, Brienza N (2008). Intra-abdominal hypertension and acute renal failure in critically ill patients. Intensive Care Med.

[27] Mohmand H, Goldfarb S (2011). Renal dysfunction associated with intra-abdominal hypertension and the abdominal compartment syndrome. J Am Soc Nephrol.

[28] Gudmundsson FF, Gislason HG, Dicko A, Horn A, Viste A, Grong K (2001). Effects of prolonged increased intra-abdominal pressure on gastrointestinal blood flow in pigs. Surg Endosc.

[29] Correa-Martín L, Castellanos G, García-Lindo M, Díaz-Güemes I, Sánchez-Margallo FM (2013). Tonometry as a predictor of inadequate splanchnic perfusion in an intra-abdominal hypertension animal model. J Surg Res.

